# Shared Curricula and Competencies in One Health and Health Professions Education

**DOI:** 10.1007/s40670-020-01140-7

**Published:** 2020-11-10

**Authors:** Roxanne J. Larsen

**Affiliations:** 1 Department of Veterinary and Biomedical Sciences, College of Veterinary Medicine, University of Minnesota, St. Paul, MN USA; 2 Medical Education Division, Duke University School of Medicine, Durham, NC USA

## Abstract

Globally, health professions education programs have similar course content and expectations for learners. One Health core competencies are shared by many health professions accreditation bodies. These competencies provide a framework which can guide professional programs in a world with emerging zoonotic diseases, a growing interface between humans and animals, and ongoing impacts from climate change. By focusing on shared outcomes, we can better prepare our learners for a more interdisciplinary practice of medicine and science. Fundamental courses, like gross anatomy, can be a uniting thread. A general overview of anatomy courses in medical and veterinary programs is provided.

Exploring the connections among human, animal, and environmental health is a key component of One Health approach (e.g., One Health Initiative, One Health Commission) [1, 2] and should be incorporated into health professions education (HPE) programs and their curricula for all levels and fields of health professional learners. The One Health approach provides a framework of core competencies including domains such as management, communication and informatics, values and ethics, leadership, team and collaboration, roles and responsibilities, and systems thinking [3, 4]. These shared competencies are especially important in guiding our health professions curricula in a world with emerging zoonotic diseases, the growing interface between humans and animals, and the ongoing impacts from climate change.

There has been a historical connection to the One Health approach in science and medicine, which contributed to the birth and development of many basic science disciplines including anatomy, physiology, and immunology (Fig. 1). The history of One Health begins as early as the time of the Egyptians and continued with several growth spurts in public health and comparative medicine. Eventually, the continued advances in medicine and technology allowed for more comparative and interventional methods to be employed [5, 6].

**Fig. 1:**
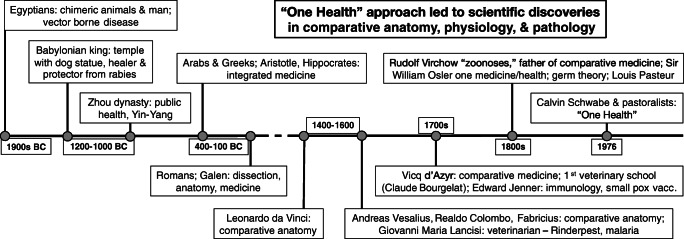
History of the One Health approach. Timeline compiled and modified from Vergis et al. [5] and Capua and Cattoli [6]

Along with a shared history, there are shared professional competencies and/or accreditation standards across a variety of health professional programs (Table 1). The programs included in this brief overview are the American Veterinary Medical Association (AVMA) Council on Education (COE) Accreditation Policies and Procedures, requirements for Standards of Accreditation (7.9. Standard 9, Curriculum) [7]; the Association of American Veterinary Medical Colleges (AAVMC) Competency-Based Veterinary Education (CBVE) competency domains and framework [8]; the Association of American Medical Colleges (AAMC) and the Liaison Committee on Medical Education (LCME) accreditation standards (Standard 7, Curricular Content) [9]; and One Health core competency domains as best summarized in Frankson et al. [3]. For brevity, the core competencies of other health profession disciplines and their accreditation bodies (e.g., osteopathic, nursing, allied health) were not included in this table, but they also emphasize similar topics. Although there are many shared competencies, we should also be asking ourselves if our current curricula provide awareness about the blending boundaries between human and animal health, and the potential impacts of global connectedness on healthcare systems. As you can see, these key features are missing from these broader curricular competencies summarized as “Common themes” in Table 1.

**Table 1:** Shared competencies and/or accreditation standards across a few representative health professions programs. Competencies and standards were gathered from the respective accreditation bodies or institutions and condensed and summarized. *AVMA COE* American Veterinary Medical Association Council on Education; *AAVMC CBVE* Association of American Veterinary Medical Colleges Competency-Based Veterinary Education; *AAMC LCME* Association of American Medical Colleges, Liaison Committee on Medical Education

AVMA COE	AAVMC CBVE	AAMC LCME	One Health	Common themes
Problem solving, theory and practice of medicine, diagnostic methods, and interpretation	Clinical reasoning, decision making, critical thinking, problem solving	Critical judgment and problem solving, diagnosis, and treatment planning	Problem solving, change makers	Critical judgment, problem solving, clinical reasoning, decision making
Contribution to teams	Collaboration, teams	IPE collaboration, teams	Teams, collaboration	IPE collaboration, teamwork
Communication	Communication	Communication	Communication	Communication
Biological principles, making and applying medical judgment	Scholarship, evidenced-based practice	Scientific knowledge, methods, and research	Systems thinking, policy, awareness of big picture	Evidenced-based practice, research, scholarship, systems thinking
Ethical and professional conduct	Professionalism, ethical reasoning, leadership	Medical ethics, values	Values, ethics, professionalism, leadership	Ethics, values, professionalism, leadership
Individual and population management	Individual and population care, welfare, management	Individual and population health	Management of teams and resources	Individual and population care, welfare, management, team/resource management
Patient health and welfare, public health	Public health, global perspective, education	Diagnosis, prevention, reporting of common society problems	Broader awareness, One Health approach, diplomacy	Awareness of healthcare disparities, public health
Diversity and inclusion	Communicates with diverse clients, colleagues, and public	Cultural competence	Diverse teams, cultural competence	Cultural competence, diversity, and inclusion
Testing and record management, treatment plan, and referral	Financial and practice management, legal and regulatory requirements			Management of records and finances, awareness of legal and regulatory requirements

As an example of a place to start developing One Health approaches early in HPE programs, I focused on anatomy courses in medical and veterinary schools. Drawing from my own experiences [10] and fellow anatomy educators [11–13], a general comparison between veterinary and medical anatomy courses outlining how anatomy is delivered, including a description of the primary educators and other instructors, is presented (Table 2). Table 2 also provides a description of the primary focus (e.g., in veterinary anatomy there is typically a semester on small animal/carnivore anatomy, and a semester on large animal/ungulate anatomy), additional concepts listed in order of emphasis, and which topics students found to be most similar or helpful in understanding the anatomy of their discipline. Student input was collected through my own experiences and observations when teaching a variety of regional anatomy courses and dissection labs, and through direct communication with students. Also noted is the observation of an increasing trend where courses focus on clinically relevant anatomy, which may be due to reductions in contact hours and the continual curricular reform and revision efforts underway at many institutions. In the end however, the similarities in HPE program curricula and the pre-clinical or basic science course content, particularly anatomy, outnumber the differences.

**Table 2:** A general overview of the delivery of anatomy and focus of anatomy content at veterinary and medical schools. This table is compiled from my own experiences as an educator [10] and from other anatomy educators [11–13]

	Veterinary schools	Medical schools
Delivery		
Primary anatomy educators are:	DVMs, PhDs (biological disciplines)	PhDs (anthropologists), MDs
Additional educators are:	Veterinary technicians, DVMs (surgeons)	Graduate students, MDs (surgeons)
Content		
Anatomy primarily focused on:	Typical pathways/patterns in carnivores and ungulates	Typical pathways/patterns in humans
Additional concepts of focus, listed in order of emphasis:	1) Vertebrate anatomy (e.g., human; domestic, agricultural, exotic animals)	1) Common variations (emphasis on surgical scenarios)
	2) Species differences	2) Clinical anatomy*
	3) Clinical anatomy*	3) Integration of structure and function
	4) Integration of structure and function	4) Vertebrate anatomy (e.g., primate, general mammal)
Students found these topics most similar:	Human anatomy helpful in understanding neuroanatomy and musculoskeletal anatomy	Vertebrate anatomy helpful in understanding neuroanatomy musculoskeletal anatomy

*Clinical anatomy is quickly becoming the primary emphasis in both

Anatomy can be used as an early platform to bring health professional learners together in an applied and meaningful way, as it is taught early in the curriculum in almost all programs. A handful of multidisciplinary universities that currently hold interprofessional education (IPE) and One Health sessions with medical, veterinary, and other health professional students include the following: Tufts University Cummings School of Veterinary Medicine and School of Medicine (Comparative Anatomy Exchange Day, An Interprofessional Tufts One Health Event), and the University of Pennsylvania School of Veterinary Medicine and Perelman School of Medicine (Penn Inter-Health School Anatomy Exchange). These types of educational activities are most often hands-on or applied sessions, and many of these interprofessional events were initiated by student clubs or organizations associated with public health, global health, or One Health. A key alliance that has helped connect these student groups globally is the International Student One Health Alliance (ISOHA) [14], which is associated with the One Health Commission [2]. Many of these clubs facilitate sessions that highlight comparative anatomy as a way to connect with other health professional students.

Educational activities meant to bring learners together should follow the general trends practiced in IPE and active learning [15], where they are (1) focused on shared concepts and experiences; (2) best carried out in small groups; (3) simple and repeatable; and (4) highlight the similarities among various health professionals. The professional traits emphasized during IPE are often already imbedded in the overarching curriculum of traditional HPE programs and also align with One Health competencies. Many may ask if these types of sessions are actually feasible. We do have limited time to deliver content, increasing demands on faculty time and effort, recommendations to reduce cognitive overload for our learners, and potentially lengthy distances between the variety of HPE programs. However, we can overcome some of these potential barriers by using technology to our advantage with tools similar to those we all are currently employing during the COVID-19 pandemic. For example, video conferencing software (e.g., Zoom, Microsoft Teams, Google Classroom) [16], video recording software (e.g., Fliprid, VoiceThread, Screencast-O-Matic), polling applications (e.g., Poll Everywhere, ChimeIn 2, Mentimeter), virtual anatomy applications (e.g., veterinary: IVALA through the Veterinary Information Network, The Glass Horse and Dog through Science in 3D; human: Visible Body, BioDigital Human), and even 3D printing of difficult to dissect structures and regions [10, 17]. Learners are relatively well equipped to navigate the new technologies and ways of communicating. We as educators now need to open ourselves up to training in these fields. We also need to embrace the healthcare models of the future. Additionally, we cannot forget that all stakeholders (including students) should be a part of the discussion if these types of events are going to be successful. Buy-in at multiple levels is key not only for the initial success of an event, but also for the sustainability of the overall concept of One Health in HPE. The intended end goal of IPE events in this context would be to allow learners from a variety of HPE programs to share knowledge, experiences, and perspectives in healthcare, so that they can be successful in meeting the ever-changing needs of a connected global population.

In sum, educators should take advantage of active-learning settings (i.e., laboratory-based course components, team-based learning); emphasize communication, collaboration, and problem-solving skills; foster an educational environment that focuses on knowledge of transdisciplinary sciences; promote awareness of a broad-based global health perspective and social and cultural aspects of health; and help develop professionals that have the skills to interact with diverse communities and can critically evaluate health policies. All of these factors highlight the need for One Health as an underlying theme in our HPE systems, so that we can all better understand the connectedness of humans, animals, and the environment. Now more than ever our developing health professionals need a shared framework that will allow them to have early and meaningful interactions with each other. These early interactions will allow them to engage and better understand diverse perspectives, which ultimately influences their abilities to provide global healthcare and practice global medicine.

## Data Availability

Not applicable
